# The Quality of Life of Seniors with Eye Diseases during COVID-19

**DOI:** 10.1155/2023/9987483

**Published:** 2023-09-26

**Authors:** Brian Edward Yu, Shehzad Ali, Hyunsoo Jang, Samantha So, Michael Huang, Cindy Hutnik, Monali Malvankar-Mehta

**Affiliations:** ^1^Schulich School of Medicine and Dentistry, The University of Western Ontario, London, ON, Canada; ^2^Department of Epidemiology and Biostatistics, Schulich School of Medicine and Dentistry, The University of Western Ontario, London, ON, Canada; ^3^Department of Ophthalmology, Schulich School of Medicine and Dentistry, The University of Western Ontario, London, ON, Canada

## Abstract

**Purpose:**

To assess health-related quality of life (HRQoL), vision-related quality of life (VRQoL), depression and anxiety symptoms, and social support and community integration of seniors with eye diseases and to identify important predictor variables of the outcomes.

**Methods:**

A cross-sectional survey was performed in seniors with eye diseases (*n* = 90). Demographic characteristics and questionnaire scores were summarized. Linear regression analysis with backward stepwise selection was used to predict the value of the outcomes of eye disease.

**Results:**

Preference-based HRQoL of the study patients with eye diseases during the pandemic was likely good with a mean utility value of 0.88. VRQoL and sleep quality appeared to be good as well. Depression and anxiety symptoms appeared to be low, while community integration and social support were moderate. Furthermore, the presence of retinal disease, number of nonocular comorbidities, and education appeared to have significant negative effects on social support and community integration. The presence of retinal disease and the number of nonocular comorbidities both appeared to negatively impact VRQoL. The use of a mobility aid appeared to negatively affect depressive symptoms and sleep quality.

**Conclusions:**

Overall quality of life and wellness among seniors with eye diseases appeared to be good during the COVID-19 pandemic. However, the presence of retinal disease and the number of nonocular comorbidities both appeared to negatively impact VRQoL and social support and community integration. Education appeared to impact social support and community integration negatively. The use of a mobility aid appeared to negatively affect depressive symptoms and sleep quality.

## 1. Introduction

Coronavirus disease 2019 (COVID-19) is a highly infectious disease caused by the severe acute respiratory syndrome coronavirus 2 (SARS-CoV-2) [1]. This disease has been reported to be able to cause severe acute respiratory infection with an incubation period of 1 to 14 days [2]. Many of the common symptoms of COVID-19 include fever, dry cough, and fatigue [3]. The SARS-CoV-2 virus has been reported to be transmitted via respiratory droplets; however, it can also be spread through various discharges, feces, aerosol, and conjunctiva [4].

The burden brought on by the COVID-19 pandemic has affected many groups of individuals on a global level. One vulnerable group of individuals who have been greatly impacted by the pandemic includes patients with ophthalmological conditions. Patients in this group are particularly vulnerable in the context of the COVID-19 pandemic because of their age and preexisting comorbid conditions; specifically, elderly patients aged 65 and above with eye diseases like glaucoma, age-related macular degeneration, or diabetic retinopathy require regular follow-ups and commonly suffer from additional comorbidities [5]. These additional conditions include anxiety and depression which have been shown to be exacerbated throughout the ongoing pandemic [6, 7]. Even if these individuals do not currently have such comorbidities, they are extremely susceptible to developing mental health issues [8].

Furthermore, the negative impact of the COVID-19 pandemic has been shown to be pronounced among elderly individuals through public media outlets portraying COVID-19 as a disease that is particularly devastating to the elderly. This spread of fearful sentiment, in turn, has resulted in the development of social stigma and discrimination causing additional distress to elderly individuals, their families, and their caregivers [8]. This information compounds their worry about being infected with the virus and not having access to proper healthcare. In addition to the vulnerabilities imposed by their age group, because of the proximity between the patient and healthcare personnel during ophthalmological examinations, the risk of transmission can be perceived as being relatively high among seniors with ophthalmologic conditions. As such, the delicate balance between the risk of exposure to COVID-19 and visual loss in delaying cases is a psychological stressor to both patients and clinicians [9, 10].

The present pandemic has led officials to rethink the management of patient lists and to restrict the patients to be assessed or treated based on the urgency of their condition in accordance with ministerial guidelines [11, 12]. These restrictions may have potentially resulted in many delays in clinical visits which, in turn, may increase the risk of visual loss not only by delaying necessary care but also by making patients less likely to follow their physician's guidance for their conditions. Moreover, due to the inability to attend clinical visits, patients themselves might decide to become nonadherent and interrupt their treatment or postpone their visit for fear of contracting SARS-CoV-2. Furthermore, the COVID-19 pandemic conditions have made it even more difficult to access medications that are high in demand and have made it difficult to make changes to treatments when necessary [13]. Additionally, visual loss can accompany depressive symptoms and deteriorate the quality of life (QoL) [14] in addition to the difficulties already present due to their ocular diseases and potential comorbidities. Thus, it is necessary to characterize and document the quality of life and mental well-being of patients with various ophthalmologic conditions during the COVID-19 pandemic.

In documenting the QoL and wellness of patients during the COVID-19 pandemic, we can quantify the collateral impact of COVID-19 beyond the direct impact of the virus. Furthermore, this will help to improve the future quality of care during non-COVID-19 conditions and even during potential future pandemic situations. Therefore, the goal of this study is to characterize the preference-based health-related quality of life (HRQoL), vision-related QoL (VRQoL), depression and anxiety symptoms, sleep quality, and social support and community integration of seniors aged 65 and above with various eye diseases during the COVID-19 pandemic. Furthermore, this study also aims to identify important predictor variables for the aforementioned measures.

## 2. Methods

### 2.1. Study Design and Sampling Procedures

The study followed a cross-sectional design. A convenience sample of 90 patients who were identified as having an underlying ocular disease was recruited from four ophthalmic practices at the Ivey Eye Institute, St. Joseph's Healthcare, London, Ontario. All patients were sequentially recruited from November 2021 to May 2022 using convenience sampling. The eligibility of the patients who were attending their regular ophthalmology visits was determined by the ophthalmologist on staff. Inclusion criteria included patients who were aged 65 and above and diagnosed with an eye disease by an experienced ophthalmologist. Exclusion criteria included patients who were unable to provide valid informed consent, who had significant communication barriers or lack of English proficiency that prevents participants from completing the questionnaires, or who had irreversible vision loss that prevented them from completing the questionnaires.

All participants received a complete explanation of the purpose and procedures involved in the study, and patient concerns were addressed prior to study participation. Verbal and electronically written informed consent was obtained from all participating patients. The study was initiated after approval by the Western University Health Science Research Ethics Board and Lawson Health Research Institute's Clinical Research Impact Committee.

### 2.2. Data Collection

All data were collected through electronic questionnaires accompanied by face-to-face interviews for assistance. All data were recorded electronically using the UWO Qualtrics questionnaires that were set up in advance. Data from Qualtrics were then imported to a password-protected and encrypted spreadsheet on the password-protected local computer in the principal investigator's (MM) office at St. Joseph's Healthcare. Data were coded to protect participant confidentiality. The code key with identifying data (master data) was also stored in a password-protected and encrypted spreadsheet on the password-protected local computer in the principal investigator's office. For analysis purposes, the deidentified study data not containing patient information were stored in a password-protected and encrypted spreadsheet on the St. Joseph's Hospital OneDrive. Data quality checks were performed at random.

Following clinical examination of the patients, the ophthalmologists at the Ivey Eye Institute identified and referred participants to the research assistant on duty based on the inclusion and exclusion criteria. The questionnaires were presented to the patients using a combination of self-administered and interviewer-assisted modes. Participants provided informed consent and completed the 30-minute questionnaire. The questionnaire included the Time Trade-Off (TTO) questionnaire, the 25-item version National Eye Institute Visual Function Questionnaire (NEI VFQ-25), Hospital Anxiety and Depression Scale–Anxiety Subscale (HADS-A), Center for Epidemiologic Studies–Depression Scale (CES-D), Pittsburgh Sleep Quality Index (PSQI), and Community Integration Questionnaire (CIQ). Demographic characteristics on patients' age, socioeconomic status (SES), ethnicity, education level, living arrangement, city of residence, and use of mobility aid were also collected, all of which were provided by patients themselves in the electronic questionnaires.

The TTO was used to obtain utility scores to calculate patients' preference-based HRQoL. Preference-based HRQoL is a frequently used measure calculated with utility values on a scale from 0 to 1, where 0 represents a health state equal to death and 1 represents a state of perfect health [15]. In the current study, the utility score using the TTO method was calculated by dividing the number of years a patient was willing to trade in return for perfect vision by the estimated number of years of life remaining and subtracting this number from 1.

VRQoL measures the impact of vision on an individual's daily living, as well as one's satisfaction and attitudes towards their vision. The NEI VFQ-25 is divided into 12 subscales: general health, general vision, near vision, distance vision, driving, peripheral vision, color vision, ocular pain, role limitations, dependency, social function, and mental health. The NEI VFQ-25 was also shown to have high validity and reliability [16].

The HADS-A is a 7-item self-report subscale for measuring symptoms of anxiety. Each item on the questionnaire is scored from 0 to 3, with total scores ranging from 0 to 21. Higher scores represent higher levels of psychological distress [17]. The HADS-A has been used in a previous study on the impact of low vision on the QoL, depression, anxiety, and social support [18]. A score ≥8 on the HADS-A has a sensitivity of 0.9 and specificity of 0.79 for identifying patients with anxiety [19]. The HADS-A has been demonstrated to have adequate internal consistency with a Cronbach's alpha value of 0.87 when administered in older adults [20].

The CES-D is a brief self-report scale designed to measure self-reported symptoms associated with depression experienced in the past week. The CES-D includes twenty items comprising six scales reflecting major facets of depression. The possible range of CES-D scores is 0 to 60, with the higher scores indicating the presence of more depressive symptomatology. A CES-D score ≥16 has high sensitivity and specificity rates for identifying subjects with depressive disorder. The CES-D has been demonstrated to be reliable with coefficient alpha estimates of 0.90 in clinical older adults. It has also been demonstrated to have high construct validity when administered to older adults [21].

The PSQI is a self-report questionnaire that assesses sleep quality over a 1-month time interval. The measure consists of 19 individual items, creating 7 components that produce one global score [22]. Higher PSQI scores indicate worse sleep quality. A PSQI score >5 has sensitivity and specificity rates of 89.6% and 86.5%, respectively, for identifying cases with sleep disorder [23]. The PSQI has been shown to have a high test-retest reliability and a good validity [24].

The CIQ is a 15-item inventory designed to measure levels of community integration. The overall score ranges from 0 to 29 and can be further divided into three subscores, corresponding to integration in the home, social integration, and productivity. A higher CIQ score represents greater integration [25]. Previous research has demonstrated adequate test-retest reliability and internal consistency [26].

All patients were interviewed under standardized conditions. The interviews were conducted by four interviewers who all received standardized training prior to administering the questionnaires. All questionnaires were completed by the patient in an electronic format on an electronic tablet through the UWO Qualtrics link containing the questionnaires. While completing the questionnaires, a research assistant was present to assist by answering any questions or concerns about the questionnaires, if patients had any, as well as to administer the questionnaires.

### 2.3. Statistical Analysis

All statistical analyses were performed using STATA 17.0. The descriptive statistics were computed for all demographic variables while univariate analysis was computed for all questionnaire outcome measures. To understand the central tendency and distribution of continuous variables, means and standard deviations were calculated.

Associations between predictor variables were also assessed using Pearson correlations between continuous predictor variables. The Pearson correlation coefficient threshold absolute value of 0.6 was used as a cutoff indicating a strong association between the variables [27]. If a threshold above 0.6 was identified, then a significance test was conducted to confirm the association. Chi-square tests were used to assess the association between pairs of categorical predictor variables. The significance of the relationship between the predictor variables was defined at *p* < 0.050. *T*-tests and one-way ANOVA were used to assess the associations between pairs of continuous and categorical predictor variables. Again, statistical significance was determined at *p* < 0.050.

Bivariate analysis was performed to assess the unadjusted effect estimates and check whether each predictor variable and outcome were associated. Each of the predictor variables (age, number of nonocular comorbidities, number of ocular comorbidities, use of a mobility aid, SES during COVID-19, living arrangements, education, presence of retinal disease, presence of glaucoma, and presence of cataracts) was individually investigated for association with all six questionnaire outcomes, using simple linear regression analyses.

Linear regression models were also created with the questionnaire scores as the dependent variables using backward stepwise multiple regression. In backward stepwise regression, all predictor variables are first used in the model. Following this, tests are then performed to determine the least significant predictor variable that is to be removed. Predictor variables continue to be removed until all remaining predictors are determined to be relevant predictors of the outcome in the model. Regression coefficients were deemed to be significant if the associated *p* values were <0.050.

To assess the backward stepwise linear regression models' abilities to accurately predict each outcome, leave-one-out cross-validation (LOOCV) of each model was performed. In LOOCV, a single observation is used for the testing set while *n* − 1 observations are used for the training set [28]. This process is repeated until each observation has been a part of the testing set. LOOCV evaluates a model based on prediction and is used for estimating the test error. The root mean square error (RMSE) and mean absolute error (MAE) were determined for each multivariable model.

Further, model assumptions for each multivariable model were tested. That is, the constant variance of the residuals was tested using residuals versus fitted value plots. The normality of the residuals was assessed using quantile-quantile (Q-Q) plots. Linearity between the predictors and outcomes was assessed using component-plus-residual plots. The variance inflation factor (VIF) was used to test for multicollinearity. A VIF of 10 was used as the rule of thumb to indicate an acceptable level of multicollinearity [29].

We believed the missing data were missing at random conditioned on the other variables (i.e., education). Then nonresponse rate was 0.06% (*n* = 5), 0% (*n* = 0), 0% (*n* = 0), 0.02% (*n* = 2), 0% (*n* = 0), and 0.02% (*n* = 2) for the TTO, NEI VFQ-25, CES-D, HADS-A, PSQI, and CIQ, respectively. Considering that the nonresponse rate was small, these individuals were excluded from all relevant analyses.

## 3. Results

### 3.1. Participants and Participant Characteristics

A total of 128 patients were approached by the attending clinicians and asked to participate in the study. Of the 128 patients, 115 agreed to participate; however, 25 of these patients did not pass the inclusion/exclusion criteria. As such, a total of 90 participants consented and were included in the study. To summarize the characteristics of these included participants, univariate analyses were conducted. The participants' characteristics can be seen in Table 1. The mean age of the participants was 77.8 years with a standard deviation of 8.0 years. In terms of the ethnicities of the participants, 86 identified as being white, two participants identified as being black, one participant identified as being Arab, and one participant did not indicate his or her ethnicity. Of the included participants, 37 participants completed high school or less and 53 participants had additional training or higher education. It was also noted that 23 participants had an income of $25,000 or less. Moreover, 67 participants lived at home with their family, spouse, or caregiver, while 22 participants lived at home alone and only one participant lived in a nursing home. Finally, 15 participants reported using a mobility aid such as a cane, walker, wheelchair, or motorized scooter. Of the 90 total participants, 65 patients reported to have other nonocular comorbidities with the most common being hypertension, hyperlipidemia, and diabetes. The mean number of nonocular comorbidities was 1.6 with a standard deviation of 1.7. Of the 77 participants who reported their eye disease(s), the mean number of ocular comorbidities was 1.2 with a standard deviation of 0.5. In terms of the eye diseases with which participants presented, 22 participants had only retinal disease, 31 participants had only glaucoma, 8 participants had only cataracts, 1 participant had only dry eye disease (DED), 1 participant had only uveitis, and 1 participant had only asteroid hyalosis. However, 3 participants had both retinal disease and glaucoma, 5 participants had both retinal disease and cataracts, and 4 participants had glaucoma and cataracts.

### 3.2. Associations between Continuous Predictor Variables

The results of the associations between continuous predictor variables are presented in Table 2. Pearson correlations did not reveal any strong linear associations between the continuous predictor variables: age, number of nonocular comorbidities, and number of ocular comorbidities.

### 3.3. Associations between Pairs of Categorical Predictor Variables

The chi-square test results of the associations between categorical predictor variables are presented in Table 3. Significant associations were observed between the presence of retinal disease and glaucoma. Among patients who had a retinal disease, 90% did not also have glaucoma. Significant associations were also observed between the presence of glaucoma and cataracts. Among patients with glaucoma, 90% did not also have cataracts.

### 3.4. Associations between Pairs of Continuous Predictor Variables

Finally, the *t*-test and one-way ANOVA results for the associations between continuous and categorical predictor variables are presented in Table 4. The use of a mobility aid was significantly associated with age, the number of nonocular comorbidities, and the number of ocular comorbidities. On average, patients who used a mobility aid were older, had more nonocular comorbidities, and had more ocular comorbidities. The presence of retinal disease was significantly associated with age. Patients who had retinal disease appeared to be older, on average. The presence of glaucoma was also significantly associated with age; however, it appeared that on average patients without glaucoma were older. Finally, the presence of cataracts was significantly associated with the number of nonocular comorbidities. On average, patients with cataracts appeared to have a greater number of nonocular comorbidities.

### 3.5. Preference-Based HRQoL

The TTO utility score measures the preference-based quality of life on a scale from 0 to 1, in which a score of 0 represents a state of death and 1 represents perfect visual health. In our study population (*n* = 90), the average TTO utility score was 0.88 with a standard deviation of 0.23 (Table 5). Thus, the majority of the patients were willing to trade 12% of their remaining life for perfect vision.

The bivariate analyses for preference-based HRQoL with the demographic and clinical variables are presented in Table 6. No variables were significantly associated with preference-based HRQoL.

On average, patients who have completed more than high school in their education have an average difference in their TTO score of 0.05 (95% CI: −0.05, 0.15) as compared to patients who have completed high school or less. The use of a mobility aid, on average, increases the TTO score by 0.02 (95% CI: −0.11, 0.16). For each increase in the number of ocular comorbidities, the TTO score increases by 0.07 (95% CI: −0.06, 0.19). On average, the presence of glaucoma increases the TTO score by 0.04 (95% CI: −0.08, 0.15). The presence of retinal disease changes the TTO score by −0.06 (95% CI: −0.17, 0.05). The presence of cataracts changes the TTO score by −0.09 (95% CI: −0.22, 0.05).

Patients with a SES of $10,001–$25,000, $25,001–$50,000, $50,001–$75,000, $75,001–$100,000, $100,001–$125,000, and greater than $150,000 are expected to have TTO scores with a difference of −0.12 (95% CI: −0.40, 0.16), −0.17 (95% CI: −0.45, 0.11), −0.06 (95% CI: −0.35, 0.22), 0.01 (95% CI: −0.31, 0.32), −0.10 (95% CI: −0.44, 0.23), and −0.33 (95% CI: −0.70, 0.04), respectively, as compared to patients with a SES of less than $10,000.

The backward stepwise regression did not produce a model with any variable predictive of the TTO utility score. This is similar to the results of the bivariate analysis in that no variable was found to be significantly associated with the TTO score.

### 3.6. VRQoL

The NEI VFQ-25 score measures vision-related quality of life on a total scale from 0 to 100, in which a score of 0 represents the worst possible score and 100 represents the best. In our study population (*n* = 90), the average NEI VFQ-25 score was 84.71 with a standard deviation of 11.61 (Table 5).

The bivariate analyses for the VRQoL with the demographic and clinical variables are presented in Table 7. The following variables were significantly associated with VRQoL: education (*p*=0.027), number of ocular comorbidities (*p*=0.042), and the presence of retinal disease. Patients who have completed more than high school in their education have an average difference in their NEI VFQ-25 score of 5.46 (95% CI: 0.62, 10.29) as compared to patients who have completed high school or less. For each increase in the number of ocular comorbidities, the NEI VFQ-25 score changes by −6.31 (95% CI: −12.23, −0.24). The presence of retinal disease changes the NEI VFQ-25 score by −7.56 (95% CI: −12.58, −2.55).

On average, for every year increase in age, the NEI VFQ-25 score changes by −0.28 (95% CI: −0.58, 0.03). Patients living at home with others are expected to have a NEI VFQ-25 score difference of 3.87 (95% CI: −1.68, 9.42) as compared to patients living alone or in a nursing/retirement home. Use of a mobility aid, on average, changes the NEI VFQ-25 score by −6.31 (95% CI: −12.83, 0.21). On average, for each increase in the number of nonocular comorbidities, the NEI VFQ-25 score changes by −1.23 (95% CI: −2.64, 0.17). On average, the presence of glaucoma increases the NEI VFQ-25 score by 2.88 (95% CI: −2.44, 8.19). The presence of cataracts increases the NEI VFQ-25 score by 1.22 (95% CI: −5.26, 7.69).

Patients with a SES of $10,001–$25,000, $25,001–$50,000, $50,001–$75,000, $75,001–$100,000, $100,001–$125,000, and greater than $150,000 are expected to have NEI VFQ-25 scores with a difference of −6.54 (95% CI: −20.58, 7.49), −4.63 (95% CI: −18.52, 9.24), −0.29 (95% CI: −14.38, 13.79), 5.53 (95% CI: −10.11, −21.17), 3.57 (95% CI: −12.46, 19.56), and −0.50 (95% CI: −17.81, 16.81), respectively, as compared to patients with a SES of less than $10,000.

Upon assessment of the backward stepwise multivariable linear regression model, the component-plus-residual plot and Q-Q plot did not confirm the assumption of linearity and normality, respectively. However, the residual versus fitted value plot and VIFs confirmed the assumption of homoscedasticity and multicollinearity, respectively (Tables 2–4). The backward stepwise multivariable regression model revealed that the presence of retinal disease and number of nonocular comorbidities were predictive of NEI VFQ-25 score (Table 8). Adjusting for the number of nonocular comorbidities, on average, the presence of retinal disease significantly (*p*=0.002) changed the NEI VFQ-25 score by −7.92 (95% CI: −12.81, −3.05). For each increase in the number of nonocular comorbidities, the NEI VFQ-25 score significantly (*p*=0.033) changes by −1.66 (95% CI: −3.01, −0.31).

### 3.7. Presence of Depressive Symptoms

The CES-D score measures self-reported symptoms associated with depression experienced in the past week on a scale from 0 to 60, in which higher scores indicate the presence of more depressive symptomatology. In our study population (*n* = 90), the average CES-D score was 6.79 with a standard deviation of 6.39 (Table 5).

The bivariate analyses for the presence of depressive symptoms with the demographic and clinical variables are presented in Table 9. The following variable was significantly associated with the presence of depressive symptoms. Use of a mobility aid, on average, increases the CES-D score by 4.35 (95% CI: 0.85, 7.86).

On average, patients who have completed more than high school in their education have an average difference in their CES-D score of 1.98 (95% CI: −0.72, 4.69) as compared to patients who have completed high school or less. Patients living at home with others are expected to have a CES-D score difference of −0.05 (95% CI: −3.13, 3.03) as compared to patients living alone or in a nursing/retirement home. For each increase in the number of ocular comorbidities, the CES-D score increases by 2.10 (95% CI: −1.33, 5.53). On average, the presence of glaucoma changes the CES-D score by −1.99 (95% CI: −4.95, 0.98). The presence of retinal disease increases the CES-D score by 2.74 (95% CI: −0.18, 5.67). The presence of cataracts changes the CES-D score by −0.43 (95% CI: −4.06, 3.20).

Patients with a SES of $10,001–$25,000, $25,001–$50,000, $50,001–$75,000, $75,001–$100,000, $100,001–$125,000, and greater than $150,000 are expected to have CES-D scores with a difference of −4.42 (95% CI: −12.61, 3.77), −3.13 (95% CI: −11.23, 4.98), −4.51 (95% CI: −12.73, 3.71), −1.24 (95% CI: −10.37, −7.89), −8.00 (95% CI: −17.35, 1.35), and −3.42 (95% CI: −13.52, 6.69), respectively, as compared to patients with a SES of less than $10,000.

Upon assessment of the backward stepwise multivariable linear regression model, Q-Q plot did not confirm the assumption of normality. However, the plot of the residual versus fitted value and the VIFs confirmed the assumption of homoscedasticity and multicollinearity, respectively (Appendix A). The backward stepwise multivariable regression model revealed that the presence of retinal disease and the use of a mobility aid were predictive of CES-D score (Table 8). On average, adjusting for the use of a mobility aid, the presence of retinal disease increased the CES-D score by 2.50 (95% CI: −0.43, 5.43); however, this increase was not significant (*p*=0.094). Adjusting for the presence of retinal disease, the use of a mobility aid significantly (*p*=0.028) increased the CES-D score by 4.20 (95% CI: 0.46, 7.94).

### 3.8. Presence of Anxiety Symptoms

The HADS-A subscale measures symptoms of anxiety in the past week each using a scale from 0 to 21 in which higher scores represent higher levels of anxiety. In our study population (*n* = 88), the average HADS-A score was 2.83 with a standard deviation of 2.56 (Table 5).

The bivariate analyses for the presence of anxiety symptoms with the demographic and clinical variables are presented in Table 10. No variables were significantly associated with the presence of anxiety symptoms.

On average, patients living at home with others are expected to have a HADS-A score difference of 0.71 (95% CI: −0.52, 1.94) as compared to patients living alone or in a nursing/retirement home. The use of a mobility aid, on average, increases the HADS-A score by 0.58 (95% CI: −0.87, 2.04). On average, for each increase in the number of nonocular comorbidities, the HADS-A score increases by 0.11 (95% CI: −0.21, 0.42). For each increase in the number of ocular comorbidities, the HADS-A score increases by 0.53 (95% CI: −0.83, 1.89). On average, the presence of glaucoma changes the HADS-A score by −0.12 (95% CI: −1.32, 1.07). The presence of retinal disease increases the HADS-A score by 0.84 (95% CI: −0.33, 2.02). The presence of cataracts changes the HADS-A score by −0.71 (95% CI: −2.15, 0.73).

Patients with a SES of $10,001–$25,000, $25,001–$50,000, $50,001–$75,000, $75,001–$100,000, $100,001–$125,000, and greater than $150,000 are expected to have HADS-A scores with a difference of −0.05 (95% CI: −3.30, 3.20), 0.21 (95% CI: −3.00, 3.42), 0.37 (95% CI: −2.89, 3.63), −1.43 (95% CI: −5.05, −2.19), −0.83 (95% CI: −4.54, 2.87), and −1.50 (95% CI: −5.51, 2.51), respectively, as compared to patients with a SES of less than $10,000.

Upon assessment of the backward stepwise linear regression model, the Q-Q plot did not confirm the assumption of normality, but the plot of the residual versus fitted value confirmed the assumption of homoscedasticity (Appendix A). The backward stepwise regression model revealed that the use of a mobility aid was the only variable predictive of HADS-A score (Table 8). Once again, this model displays that, on average, the use of a mobility aid increases the HADS-A score by 0.58 (95% CI: −0.87, 2.04); however, this change is not significant (*p*=0.428).

### 3.9. Sleep Quality

The PSQI score measures sleep quality over a 1-month time interval on a global scale from 0 to 21, in which higher scores indicate worse sleep quality. In our study population (*n* = 90), the average PSQI score was 6.58 with a standard deviation of 3.00 (Table 5).

The bivariate analyses for sleep quality with the demographic and clinical variables are presented in Table 11. The following variables were significantly associated with sleep quality: use of a mobility aid (*p*=0.044) and SES during COVID-19. The use of a mobility aid, on average, increases the PSQI score by 1.73 (95% CI: 0.05, 3.41). Patients with a SES of $10,001–$25,000, $25,001–$50,000, $50,001–$75,000, $75,001–$100,000, $100,001–$125,000, and greater than $150,000 are expected to have PSQI scores with a difference of −3.26 (95% CI: −7.00, 0.48), −3.26 (95% CI: −6.95, 0.43), −3.47 (95% CI: −7.21, 0.26), −4.43 (95% CI: −8.58, −0.28), −4.67 (95% CI: −8.92, −0.41), and −2.50 (95% CI: −7.10, 2.10), respectively, as compared to patients with a SES of less than $10,000.

On average, for every year increase in age, the PSQI score changes by −0.01 (95% CI: −0.09, 0.08). Patients who have completed more than high school in their education have an average difference in their PSQI score of 0.82 (95% CI: −0.48, 2.11) as compared to patients who have completed high school or less. Patients living at home with others are expected to have a PSQI score difference of −1.17 (95% CI: −2.62, 0.29) as compared to patients living alone or in a nursing/retirement home. On average, for each increase in the number of nonocular comorbidities, the PSQI score increases by 0.22 (95% CI: −0.15, 0.59). For each increase in the number of ocular comorbidities, the PSQI score increases by 0.36 (95% CI: −1.18, 1.90). On average, the presence of glaucoma changes the PSQI score by −0.56 (95% CI: −1.94, 0.81). The presence of retinal disease increases the PSQI score by 0.57 (95% CI: −0.82, 1.95). Finally, the presence of cataracts changes the PSQI score by −0.28 (95% CI: −1.97, 1.42).

Upon assessment of the backward stepwise linear regression model, the Q-Q plot did not confirm the assumption of normality, but the plot of the residual versus fitted value confirmed the assumption of homoscedasticity (Appendix A). The backward stepwise regression model revealed that the use of a mobility aid was the only variable predictive of PSQI score (Table 8). Once again, this model displays that, on average, the use of a mobility aid significantly (*p*=0.044) increases the PSQI score by 1.73 (95% CI: 0.05, 3.41).

### 3.10. Social Support and Community Integration

The CIQ score measures social support and community integration on a scale from 0 to 29, in which a higher score represents more complete community integration and a higher level of social support. In our study population (*n* = 88), the average CIQ total score was 14.46 with a standard deviation of 4.07 (Table 5).

The bivariate analyses for social support and community integration with the demographic and clinical variables are presented in Table 12. The following variables were significantly associated with social support and community integration: number of nonocular comorbidities (*p*=0.047) and presence of retinal disease (*p*=0.001). On average, for each increase in the number of nonocular comorbidities, the CIQ score changes by −0.50 (95% CI: −1.00, −0.01). The presence of retinal disease changes the CIQ score by −3.06 (95% CI: −4.82, −1.30).

On average, for every year increase in age, the CIQ score changes by −0.10 (95% CI: −0.21, 0.02). Patients who have completed more than high school in their education have an average difference in their CIQ score of −1.23 (95% CI: −2.98, 0.53) as compared to patients who have completed high school or less. Patients living at home with others are expected to have a CIQ score difference of −0.67 (95% CI: −2.67, 1.33) as compared to patients living alone or in a nursing/retirement home. Use of a mobility aid, on average, changes the CIQ score by −1.22 (95% CI: −3.54, −1.10). The presence of glaucoma increases the CIQ score by 0.80 (95% CI: −1.14, 2.73). The presence of cataracts changes the CIQ score by −0.07 (95% CI: −2.45, 2.31). On average, for each increase in the number of ocular comorbidities, the CIQ score changes by −1.51 (95% CI: −3.70, 0.68).

Patients with a SES of $10,001–$25,000, $25,001–$50,000, $50,001–$75,000, $75,001–$100,000, $100,001–$125,000, and greater than $150,000 are expected to have CIQ scores with a difference of −0.02 (95% CI: −5.30, 5.25), 0.33 (95% CI: −4.88, −5.54), 0.13 (95% CI: −5.15, 5.40), 3.38 (95% CI: −2.48, 9.24), 0.92 (95% CI: −5.09, 6.92), and 1.67 (95% CI: −4.82, 8.15), respectively, as compared to patients with a SES of less than $10,000.

Upon assessment of the backward stepwise multivariable linear regression model, the component-plus-residual plot, Q-Q plot, residual versus fitted value plot, and the VIFs confirmed the assumption of linearity, normality, homoscedasticity, and multicollinearity, respectively (Appendix A). The backward stepwise multivariable regression model revealed that the presence of retinal disease, number of nonocular comorbidities, and education were predictive of CIQ score (Table 8). Adjusting for all other predictors, on average, the presence of retinal disease significantly (*p* < 0.001) changed the CIQ score by −3.10 (95% CI: −4.77, −1.43). For each increase in the number of nonocular comorbidities, the CIQ score significantly (*p*=0.004) changes by −0.68 (95% CI: −1.15, −0.22). Finally, having an education of more than high school significantly (*p*=0.033) changes the CIQ score on average by −1.79 (95% CI: −3.44, −0.15).

### 3.11. Results of the LOOCV

The results of LOOCV assessment of the models in Table 13 demonstrate that the backward stepwise regression models for VRQoL and social support and community integration had generally similar MAEs as compared to a similar previous study by Uruthiramoorthy et al. [30]. The MAE of the VRQoL model in the current study is slightly higher than the models in the previous study suggesting that the model in the current study could be nearly as predictive as the previous models. However, the multivariable model for social support and community integration in the current study had an even lower value of MAE as compared to the study by Uruthiramoorthy et al. [30] indicating that it may be more predictive of the outcome.

## 4. Discussion

In this cross-sectional study, the preference-based HRQoL, VRQoL, depression and anxiety symptoms, and social support and community integration of seniors with various eye diseases were assessed. A total of 90 participants were included in the study, and the results showed that the COVID-19 pandemic did not appear to have a large impact on the QoL of seniors with eye diseases. This section provides a detailed analysis and discussion of the results of the QoL assessment and the potential impact on health-related outcomes.

The present study revealed that the preference-based HRQoL of our study patients with eye diseases during the pandemic is likely quite good with a mean utility value of 0.88. Moreover, it does not appear that any of the potential predictors had a significant level of impact on the HRQoL of seniors during the pandemic. The analysis of the present study also showed that these seniors with eye disease appeared to have good vision and only a slight loss in visual performance impacting their VRQoL during the pandemic. However, it was also shown that the presence of retinal disease and the number of nonocular comorbidities had a significant negative impact on the participants' VRQoL.

Moreover, study participants appeared to have a low presence of depressive symptomatology. The backward stepwise multivariable regression analysis revealed that the use of a mobility aid did appear to show a significant increase in depressive symptomatology. The level of anxiety appeared to be normal, and the quality of sleep appeared to be good overall among patients with eye diseases during the pandemic. Of note, the use of a mobility aid was found to negatively affect sleep quality but not the presence of anxiety symptoms. Previously, sleep quality has been found to be associated with physical disability in older adults which may explain this relationship between the use of mobility aid and poor sleep quality [31]. On the other hand, an analysis of the levels of social support and community integration found that participants likely had moderate social support and community integration during the pandemic. We speculate that this finding was a result of those with a higher education had a better understanding of the pandemic conditions and consequences. This higher education and better understanding likely then led individuals to socially isolate more and avoid others which, in turn, had a negative impact on their social support and community integration. We also speculate that through this mechanism, as a result of poorer social support and community integration, both HRQoL and VRQoL may have been negatively impacted (albeit not significantly). Furthermore, it was revealed that the presence of retinal disease, number of nonocular comorbidities, and education appeared to have significant negative effects on social support and community integration. Based on the key measures of QoL such as preference-based HRQoL, VRQoL levels of depression and anxiety, and access to social support and community integration, the above findings indicate that the QoL and wellness of the elderly with eye diseases appear to be good.

However, it is important to note that there are studies conducted during this period that highlighted the need for further research to ascertain the impact of COVID-19 on the QoL of the elderly with eye diseases. For instance, a systematic review by Zaher et al. [32] showed that there was a 36% increase in the fear of vision loss due to an increased risk of missing appointments and a 48% increase in the fear of contracting the virus due to office visits. Therefore, a future survey study focusing on gathering data about the fear of vision loss due to an increased risk of missing appointments and fear of contracting the virus due to office visits among eye disease patients would shed light on the overall QoL of patients during the ongoing pandemic. Further, a prospective cross-sectional comparative study by Shalaby et al. [33] found that the pandemic affected visually impaired people which results in diminished QoL. Moreover, majority of seniors who have vision loss tend to experience depressive symptoms [34]. The results of our study resonate that community integration and social support appear to be the lowest among the study population. This finding concurs with the view that containment measures of the pandemic such as lockdowns, social distancing, and curfews may have potentially impacted the lives of seniors with eye diseases.

Interestingly, the results of the preference-based HRQoL in this current study seem to agree with those of previous studies performed in the same location and setting. A previous study by Thomas et al. [35] investigated the preference-based HRQoL among patients with glaucoma and diabetic retinopathy. Another previous study by Uruthiramoorthy et al. [30] investigated the preference-based HRQoL among patients with glaucoma. Both studies reported relatively high mean utility values from 0.89 to 0.94 which is like the value of 0.88 found in the current study using the same TTO technique. These similarities may be suggestive of the fact that perhaps the QoL of the patients at this location is relatively high due to the services and facilities provided by the Ivey Eye Institute or due to the environment of London, Ontario, in general.

Furthermore, the study by Uruthiramoorthy et al. [30] found that living arrangements and the use of a mobility aid were significant predictors of VRQoL. Additionally, the study by Uruthiramoorthy et al. [30] found age, sex, income, living arrangement, and use of mobility aids to be predictors of social support and community integration. The difference seen between the predictor variables in the two studies may be due to a multitude of factors such as the COVID-19 pandemic conditions, different time periods, general eye disease patients versus glaucoma only patients, or the senior patient population.

While the current study provides valuable information on the QoL of seniors with eye diseases, there were some limitations. A potential limitation stems from the use of convenience sampling which is associated with sampling bias [36]. Of note, many participants in our study were identified as being white and not requiring the use of a mobility aid, both of which are characteristics associated with a higher QoL [37, 38]. Moreover, the mean number of nonocular comorbidities was relatively low at 1.6, which may also have been associated with a higher QoL. As a result, this means that the results may not be representative of the actual population and that they are likely not entirely generalizable to other populations of seniors with eye diseases [39].

This study was also limited by the number of included participants. As with most studies, more participants would allow for greater power and a greater ability to detect differences that are present. As such, a greater number of participants may have resulted in the detection of a significant impact on outcome measures like preference-based HRQoL. Additionally, there is an absence of data referring to the present ocular diseases such as the visual acuity of the participants which may have influenced HRQoL or VRQoL. Furthermore, participants were divided into more general subgroups regarding their ocular diseases without specifying the disease to maintain numbers within groups for data analysis. However, this may also have influenced the perceived quality of life and the impact of the pandemic.

Two other limitations of this study are inherent to the cross-sectional design that was used. The first of which is that in a cross-sectional design one cannot necessarily determine whether the exposure did precede the outcome [40]. As such, in the current study, it is not entirely certain as to whether the study participants' wellness was due to the presence of the COVID-19 pandemic conditions. The second of which is length bias which is systematic error due to selection of disproportionate number of long duration cases [40]. With this limitation in mind, it may be that many participants recruited in the current study were patients who have had their eye diseases for a long duration of time and been properly managed for a long time as well. As such, these patients may have been more adapted to their conditions and reported better QoL and wellness.

The final potential limitation of the study stems from healthy volunteer bias. Historically, volunteers in medical research tend to have a lower risk of mortality and other health problems compared to those who are not volunteers [41]. Patients in the current study participated on a voluntary basis. As a result, this may mean that the results of the current study were biased in favor of higher QoL due to the study participants having healthier lifestyles than those who did not volunteer to participate. However, it was noted that of the 128 patients who were approached, 115 agreed to participate while 25 of these patients did not pass the inclusion/exclusion criteria. This suggests that the probability of such selection bias is likely low.

The current study has major implications, especially when it comes to focusing healthcare resources on the most vulnerable groups of the population. However, future studies in this area could focus on the relationship between QoL and eye diseases using eye disease-specific questionnaires. For example, instruments such as the Ocular Surface Disease Index for patients with DED and the Glaucoma Quality of Life-15 for patients with glaucoma could be studied [42, 43]. Generic preference-based measures of health to measure HRQoL such as the EuroQoL-5D and 36-Item Short Form Survey could be used [44, 45]. Moreover, future studies with larger sample sizes focused on the QoL of seniors with eye diseases after the pandemic is completely over would be worthwhile to conduct so that a comparison can be made to better understand the impact of the pandemic conditions on this population of patients.

## 5. Conclusions

Overall, the current study has found that the QoL among seniors with eye diseases appeared to be good. Measures of preference-based HRQoL and VRQoL appeared to be high. Indications of depression and anxiety symptoms were likely low, while community integration and social support appeared to be moderate. The presence of retinal disease and the number of nonocular comorbidities both appeared to negatively impact VRQoL and social support and community integration. Education appeared to impact CIQ negatively. The use of a mobility aid appeared to negatively affect depressive symptoms and sleep quality.

## Figures and Tables

**Table 1:** Demographic characteristics of the included participants.

Characteristics	Full sample (*N* = 90)
Age, mean (SD)	77.8 (8.0)

*Ethnicity, n (%)*	
White	86/90 (96%)
Black	2/90 (2%)
Arab	1/90 (1%)
Choice not listed	1/90 (1%)

*Education, n (%)*	
Some high school or less	8/90 (9%)
Completed high school	29/90 (32%)
Additional training	15/90 (17%)
College degree	18/90 (20%)
Undergraduate university	9/90 (10%)
Postgraduate university	8/90 (9%)
Advanced professional degree	3/90 (3%)

*Socioeconomic status, n (%)*	
Less than $10,000	3/83 (4%)
$10,001–$25,000	20/83 (24%)
$25,001–$50,000	24/83 (29%)
$50,001–$75,000	19/83 (23%)
$75,001–$100,000	7/83 (8%)
$100,001–$125,000	6/83 (7%)
$125,001–$150,000	0/83 (0%)
Greater than $150,000	4/83 (5%)

*Use of a mobility aid, n (%)*	
No	72/87 (83%)
Yes	15/87 (17%)

*Living arrangements, n (%)*	
Nursing home	1/90 (1%)
Home alone	22/90 (24%)
Home with caregiver	1/90 (1%)
Home with spouse	58/90 (64%)
Home with family	8/90 (9%)

*City of residence, n (%)*	
London	51/88 (58%)
St. Thomas	6/88 (6%)
Stratford	4/88 (5%)
Woodstock	2/88 (4%)
Rural	25/88 (29%)

*Eye diseases, n (%)*	
None reported	13/90 (18%)
Retinal only	22/90 (21%)
Glaucoma only	31/90 (34%)
Cataracts only	8/90 (9%)
Dry eye only	1/90 (1%)
Retinal and glaucoma	3/90 (3%)
Retinal and cataracts	5/90 (6%)
Glaucoma and cataracts	4/90 (4%)
Glaucoma and dry eye	1/90 (1%)
Uveitis only	1/90 (1%)
Asteroid hyalosis only	1/90 (1%)

Number of ocular comorbidities, mean (SD)	1.2 (0.5)

Number of nonocular comorbidities, mean (SD)	1.6 (1.7)

**Table 2:** Pearson correlation coefficients (*p* values) for the associations between continuous predictor variables.

	Age	Number of nonocular comorbidities	Number of ocular comorbidities
Age	1.00		
Number of nonocular comorbidities	−0.05	1.00	
Number of ocular comorbidities	0.09	0.27	1.00

**Table 3:** Chi-square tests (*p* value) for the associations between categorical predictor variables.

	Education	Living arrangements	Socioeconomic status during COVID-19	Use of a mobility aid	Retinal disease	Glaucoma	Cataracts
Education		0.448	0.382	0.203	0.817	0.850	0.567
Living arrangements			0.800	0.360	0.941	0.718	0.088
Socioeconomic status during COVID-19				0.583	0.870	0.801	0.116
Use of a mobility aid					0.087	0.720	0.647
Retinal disease						<0.001	0.386
Glaucoma							0.010
Cataracts							

**Table 4:** Results of associations between continuous and categorical predictor variables.

	Age	Number of nonocular comorbidities	Number of ocular comorbidities
	*p* value	*p* value	*p* value
	Mean (SD)	Mean (SD)	Mean (SD)
Education	0.550	0.254	0.835
Completed high school or less	78.41 (8.29)	1.84 (1.83)	1.16 (0.45)
Completed more than high school	77.38 (7.79)	1.42 (1.63)	1.18 (0.44)
Living arrangements	0.372	0.237	0.330
Home alone/nursing/retirement home	79.09 (8.77)	1.96 (1.92)	1.09 (0.29)
Home with others	77.36 (7.70)	1.46 (1.65)	1.20 (0.49)
Socioeconomic status during COVID-19	0.319	0.125	0.671
Less than $10,000	72.33 (3.79)	0.33 (0.58)	1.00 (0)
$10,001–$25,000	77.55 (7.03)	2.30 (1.75)	1.29 (0.69)
$25,001–$50,000	79.08 (9.36)	1.29 (1.46)	1.13 (0.34)
$50,001–$75,000	74.42 (6.25)	1.58 (1.50)	1.00 (0)
$75,001–$100,000	78.00 (4.24)	1.29 (1.98)	1.17 (0.41)
$100,001–$125,000	81.33 (10.42)	1.67 (1.21)	1.25 (0.50)
$125,001–$150,000			
Greater than $150,000	79.50 (7.94)	0.25 (0.50)	1.25 (0.50)
Use of a mobility aid	0.027	0.005	0.006
No	77.13 (7.73)	1.32 (1.47)	1.10 (0.35)
Yes	82.13 (8.20)	2.60 (2.03)	1.46 (0.66)
Retinal disease	0.001	0.585	0.973
No	75.61 (6.20)	1.69 (1.82)	1.17 (0.48)
Yes	81.87 (9.66)	1.47 (1.61)	1.17 (0.38)
Glaucoma	0.038	0.348	0.078
No	79.84 (9.10)	1.50 (1.59)	1.08 (0.27)
Yes	76.00 (6.81)	1.88 (1.90)	1.26 (0.55)
Cataracts	0.532	0.025	0.051
No	78.18 (8.84)	1.46 (1.65)	1.12 (0.37)
Yes	76.76 (5.27)	2.53 (1.91)	1.35 (0.61)

**Table 5:** Summary of questionnaire scores for all participants.

Questionnaire	Mean score (SD)	Minimum score	Maximum score	Number of respondents
TTO	0.88 (0.23)	0	1.00	85
NEI VFQ-25	84.71 (11.61)	54.72	98.33	90
CES-D	6.79 (6.39)	0	27.00	90
PSQI	6.58 (3.00)	2.00	15.00	88
HADS-A	2.83 (2.56)	0	12.00	90
CIQ	14.46 (4.07)	2.00	23.75	88

TTO: Time Trade-Off; NEI VFQ-25: National Eye Institute 25-Item Visual Function Questionnaire; CES-D: Center for Epidemiologic Studies Depression Scale; PSQI: Pittsburg Sleep Quality Index; HADS-A: Hospital Anxiety and Depression Scale–Anxiety; CIQ: Community Integration Questionnaire.

**Table 6:** Unadjusted effects of variables with Time Trade-Off preference-based HRQoL.

Variables	Coefficient	*P* value
Age	−0.00	0.840
Education		
Completed high school or less	Ref	
Completed more than high school	0.05	0.327
Living arrangement		
Home alone/nursing/retirement home	Ref	
Home with others	−0.01	0.854
Use of mobility aid		
Does not use mobility aid	Ref	
Uses mobility aid	0.02	0.740
Number of nonocular comorbidities	0.01	0.399
Number of ocular comorbidities	0.07	0.305
Glaucoma		
No	Ref	
Yes	0.04	0.516
Retinal disease		
No	Ref	
Yes	−0.06	0.281
Cataract		
No	Ref	
Yes	−0.09	0.195
Socioeconomic status during COVID-19		
Less than $10,000	Ref	
$10,001–$25,000	−0.12	0.406
$25,001–$50,000	−0.17	0.231
$50,001–$75,000	−0.06	0.658
$75,001–$100,000	0.01	0.966
$100,001–$125,000	−0.10	0.535
$125,001–$150,000		
Greater than $150,000	−3.27	0.084

**Table 7:** Unadjusted effects of variables with VRQoL.

Variables	Coefficient	*P* value
Age	−0.28	0.073
Education		
Completed high school or less	Ref	
Completed more than high school	5.46	0.027
Living arrangement		
Home alone/nursing/retirement home	Ref	
Home with others	3.87	0.169
Use of mobility aid		
Does not use mobility aid	Ref	
Uses mobility aid	−6.31	0.058
Number of nonocular comorbidities	−0.00	0.994
Number of ocular comorbidities	−6.24	0.042
Glaucoma		
No	Ref	
Yes	2.88	0.284
Retinal disease		
No	Ref	
Yes	−7.57	0.004
Cataract		
No	Ref	
Yes	1.22	0.709
Socioeconomic status during COVID-19		
Less than $10,000	Ref	
$10,001–$25,000	−6.54	0.356
$25,001–$50,000	−4.64	0.508
$50,001–$75,000	−0.29	0.967
$75,001–$100,000	5.53	0.484
$100,001–$125,000	3.57	0.659
$125,001–$150,000		
Greater than $150,000	−0.50	0.954

**Table 8:** Coefficient estimates (95% confidence interval) for the backward stepwise linear regression models of the questionnaire outcomes.

Variables	NEI VFQ-25 model	CES-D model	PSQI model	HADS-A model	CIQ model
Retinal disease	−7.92 (−12.81, −3.05)	2.50 (−0.43, 5.43)	**—**	**—**	−3.10 (−4.77, −1.43)
Number of nonocular comorbidities	−1.66 (−3.01, −0.31)	**—**	**—**	**—**	−0.68 (−1.15, −0.22)
Use of a mobility aid	**—**	4.20 (0.46, 7.94)	1.73 (0.05, 3.41)	0.58 (−0.87, 2.04)	**—**
Education	**—**	**—**	**—**	**—**	−1.79 (−3.44, −0.15)

NEI VFQ-25: National Eye Institute 25-Item Visual Function Questionnaire; CES-D: Center for Epidemiologic Studies Depression Scale; PSQI: Pittsburg Sleep Quality Index; HADS-A: Hospital Anxiety and Depression Scale–Anxiety; CIQ: Community Integration Questionnaire; —: variable was not included in the model.

**Table 9:** Unadjusted effects of variables with depressive symptoms.

Variables	Coefficient	*P* value
Age	−0.01	0.918
Education		
Completed high school or less	Ref	
Completed more than high school	1.98	0.149
Living arrangement		
Home alone/nursing/retirement home	Ref	
Home with others	−0.05	0.974
Use of mobility aid		
Does not use mobility aid	Ref	
Uses mobility aid	4.35	0.016
Number of nonocular comorbidities	−0.00	0.994
Number of ocular comorbidities	2.10	0.227
Glaucoma		
No	Ref	
Yes	−1.99	0.186
Retinal disease		
No	Ref	
Yes	2.74	0.066
Cataract		
No	Ref	
Yes	−0.43	0.812
Socioeconomic status during COVID-19		
Less than $10,000	Ref	
$10,001–$25,000	−4.42	0.286
$25,001–$50,000	−3.13	0.445
$50,001–$75,000	−4.51	0.278
$75,001–$100,000	−1.24	0.788
$100,001–$125,000	−8.00	0.093
$125,001–$150,000		
Greater than $150,000	−3.42	0.503

**Table 10:** Unadjusted effects of variables with anxiety symptoms.

Variables	Coefficient	*P* value
Age	−0.01	0.688
Education		
Completed high school or less	Ref	
Completed more than high school	−0.01	0.989
Living arrangement		
Home alone/nursing/retirement home	Ref	
Home with others	0.71	0.253
Use of mobility aid		
Does not use mobility aid	Ref	
Uses mobility aid	0.58	0.428
Number of nonocular comorbidities	0.11	0.492
Number of ocular comorbidities	0.53	0.438
Glaucoma		
No	Ref	
Yes	−0.12	0.838
Retinal disease		
No	Ref	
Yes	0.84	0.157
Cataract		
No	Ref	
Yes	−0.71	0.328
Socioeconomic status during COVID-19		
Less than $10,000	Ref	
$10,001–$25,000	−0.05	0.976
$25,001–$50,000	0.21	0.898
$50,001–$75,000	0.37	0.822
$75,001–$100,000	−1.43	0.434
$100,001–$125,000	−0.83	0.656
$125,001–$150,000		
Greater than $150,000	−1.50	0.458

**Table 11:** Unadjusted effects of variables with sleep quality.

Variables	Coefficient	*P* value
Age	−0.01	0.831
Education		
Completed high school or less	Ref	
Completed more than high school	0.82	0.212
Living arrangement		
Home alone/nursing/retirement home	Ref	
Home with others	−1.17	0.115
Use of mobility aid		
Does not use mobility aid	Ref	
Uses mobility aid	1.73	0.044
Number of nonocular comorbidities	0.22	0.239
Number of ocular comorbidities	0.36	0.647
Glaucoma		
No	Ref	
Yes	−0.56	0.417
Retinal disease		
No	Ref	
Yes	0.57	0.416
Cataract		
No	Ref	
Yes	−0.28	0.747
Socioeconomic status during COVID-19		
Less than $10,000	Ref	
$10,001–$25,000	−3.26	0.086
$25,001–$50,000	−3.26	0.083
$50,001–$75,000	−3.47	0.068
$75,001–$100,000	−4.43	0.037
$100,001–$125,000	−4.67	0.032
$125,001–$150,000		
Greater than $150,000	−2.50	0.282

**Table 12:** Unadjusted effects of variables with social support and community integration.

Variables	Coefficient	*P* value
Age	−0.10	0.092
Education		
Completed high school or less	Ref	
Completed more than high school	−1.23	0.169
Living arrangement		
Home alone/nursing/retirement home	Ref	
Home with others	−0.67	0.509
Use of mobility aid		
Does not use mobility aid	Ref	
Uses mobility aid	−1.22	0.297
Number of nonocular comorbidities	−0.50	0.047
Number of ocular comorbidities	−1.51	0.173
Glaucoma		
No	Ref	
Yes	0.80	0.415
Retinal disease		
No	Ref	
Yes	−3.06	0.001
Cataract		
No	Ref	
Yes	−0.07	0.951
Socioeconomic status during COVID-19		
Less than $10,000	Ref	
$10,001–$25,000	−0.02	0.993
$25,001–$50,000	0.33	0.900
$50,001–$75,000	0.13	0.962
$75,001–$100,000	3.38	0.254
$100,001–$125,000	0.92	0.762
$125,001–$150,000		
Greater than $150,000	1.67	0.610

**Table 13:** Backward linear regression model assessments from leave-one-out cross-validation.

Model outcome	Root mean square error	Mean absolute error
NEI VFQ-25	110	103
CES-D	223	214
PSQI	197	120
HADS-A	134	121
CIQ	202	210

NEI VFQ-25: National Eye Institute 25-Item Visual Function Questionnaire; CES-D: Center for Epidemiologic Studies Depression Scale; PSQI: Pittsburg Sleep Quality Index; HADS-A: Hospital Anxiety and Depression Scale–Anxiety; CIQ: Community Integration Questionnaire.

## Data Availability

The data used to support the findings of this study are available from the corresponding author upon request.

## Supplementary Materials

Supplementary Materials Appendix A: results of the validation of backward stepwise linear regression models.
