# Supplementary material for: The Quality of Life of Seniors with Eye Diseases during COVID-19
Source: J Ophthalmol. 2023 Sep 26;2023:9987483. doi: 10.1155/2023/9987483 (PMC11390238; doi:10.1155/2023/9987483)
Supplement: Supplementary Materials — Appendix A: results of the validation of backward stepwise linear regression models. [file 9987483.f1.docx]

**Appendix A.** Results of the Associations Between Predictor Variables

Pearson correlation coefficients (p-values) for the associations between continuous

predictor variables.

|  | **Age** | **Number of Non-ocular Comorbidities** | **Number of Ocular Comorbidities** |
| --- | --- | --- | --- |
| **Age** | 1.00 |  |  |
| **Number of Non-ocular Comorbidities** | -0.05 | 1.00 |  |
| **Number of Ocular Comorbidities** | 0.09 | 0.27 | 1.00 |

Chi-square tests (p-value) for the associations between categorical predictor variables.

|  | **Education** | **Living arrangements** | **Socioeconomic status during COVID-19** | **Use of a mobility aid** | **Retinal disease** | **Glaucoma** | **Cataracts** |
| --- | --- | --- | --- | --- | --- | --- | --- |
| **Education** |  | 0.448 | 0.382 | 0.203 | 0.817 | 0.850 | 0.567 |
| **Living arrangements** |  |  | 0.800 | 0.360 | 0.941 | 0.718 | 0.088 |
| **Socioeconomic status during COVID-19** |  |  |  | 0.583 | 0.870 | 0.801 | 0.116 |
| **Use of a mobility aid** |  |  |  |  | 0.087 | 0.720 | 0.647 |
| **Retinal disease** |  |  |  |  |  | <0.001 | 0.386 |
| **Glaucoma** |  |  |  |  |  |  | 0.010 |
| **Cataracts** |  |  |  |  |  |  |  |

Results of associations between continuous and categorical predictor variables.

|  | **Age**  *p-value*  *mean (SD)* | **Number of Non-ocular Comorbidities**  *p-value*  *mean (SD)* | **Number of Ocular Comorbidities**  *p-value*  *mean (SD)* |
| --- | --- | --- | --- |
| **Education** | 0.550 | 0.254 | 0.835 |
| Completed high school or less | 78.41 (8.29) | 1.84 (1.83) | 1.16 (0.45) |
| Completed more than high school | 77.38 (7.79) | 1.42 (1.63) | 1.18 (0.44) |
| **Living arrangements** | 0.372 | 0.237 | 0.330 |
| Home alone/ Nursing/Retirement home | 79.09 (8.77) | 1.96 (1.92) | 1.09 (0.29) |
| Home with others | 77.36 (7.70) | 1.46 (1.65) | 1.20 (0.49) |
| **Socioeconomic status during COVID-19**  less than $10,000  $10,001 - $25,000  $25,001 - $50,000  $50,001 - $75,000  $75,001 - $100,000  $100,001 - $125,000  $125,001 - $150,000  Greater than $150,000 | 0.319  72.33 (3.79)  77.55 (7.03)  79.08 (9.36)  74.42 (6.25)  78.00 (4.24)  81.33 (10.42)  79.50 (7.94) | 0.125  0.33 (0.58)  2.30 (1.75)  1.29 (1.46)  1.58 (1.50)  1.29 (1.98)  1.67 (1.21)  0.25 (0.50) | 0.671  1.00 (0)  1.29 (0.69)  1.13 (0.34)  1.00 (0)  1.17 (0.41)  1.25 (0.50)  1.25 (0.50) |
| **Use of a mobility aid**  No  Yes | 0.027  77.13 (7.73)  82.13 (8.20) | 0.005  1.32 (1.47)  2.60 (2.03) | 0.006  1.10 (0.35)  1.46 (0.66) |
| **Retinal disease** | 0.001 | 0.585 | 0.973 |
| No | 75.61 (6.20) | 1.69 (1.82) | 1.17 (0.48) |
| Yes | 81.87 (9.66) | 1.47 (1.61) | 1.17 (0.38) |
| **Glaucoma** | 0.038 | 0.348 | 0.078 |
| No | 79.84 (9.10) | 1.50 (1.59) | 1.08 (0.27) |
| Yes | 76.00 (6.81) | 1.88 (1.90) | 1.26 (0.55) |
| **Cataracts** | 0.532 | 0.025 | 0.051 |
| No | 78.18 (8.84) | 1.46 (1.65) | 1.12 (0.37) |
| Yes | 76.76 (5.27) | 2.53 (1.91) | 1.35 (0.61) |

**Appendix B.** Results of the Bivariate Linear Regression Analyses

Unadjusted effects of variables with time trade-off preference based HRQoL.

| **Variables** | **Coefficient** | **P-value** |
| --- | --- | --- |
| **Age** | -0.00 | 0.840 |
| **Education** |  |  |
| Completed high school or less | *Ref* |  |
| Completed more than high school | 0.05 | 0.327 |
| **Living Arrangement** |  |  |
| Home alone/ Nursing/Retirement home | *Ref* |  |
| Home with others | -0.01 | 0.854 |
| **Use of Mobility Aid** |  |  |
| Does not use mobility aid | *Ref* |  |
| Uses mobility aid | 0.02 | 0.740 |
| **Number of Non-ocular Comorbidities** | 0.01 | 0.399 |
| **Number of Ocular Comorbidities** | 0.07 | 0.305 |
| **Glaucoma** |  |  |
| No | *Ref* |  |
| Yes | 0.04 | 0.516 |
| **Retinal disease** |  |  |
| No | *Ref* |  |
| Yes | -0.06 | 0.281 |
| **Cataract** |  |  |
| No | *Ref* |  |
| Yes | -0.09 | 0.195 |
| **Socioeconomic status during COVID-19** |  |  |
| less than $10,000 | *Ref* |  |
| $10,001 - $25,000 | -0.12 | 0.406 |
| $25,001 - $50,000 | -0.17 | 0.231 |
| $50,001 - $75,000 | -0.06 | 0.658 |
| $75,001 - $100,000 | 0.01 | 0.966 |
| $100,001 - $125,000 | -0.10 | 0.535 |
| $125,001 - $150,000 |  |  |
| Greater than $150,000 | -3.27 | 0.084 |

Unadjusted effects of variables with VRQoL.

| **Variables** | **Coefficient** | **P-value** |
| --- | --- | --- |
| **Age** | -0.28 | 0.073 |
| **Education** |  |  |
| Completed high school or less | *Ref* |  |
| Completed more than high school | 5.46 | **0.027** |
| **Living Arrangement** |  |  |
| Home alone/ Nursing/Retirement home | *Ref* |  |
| Home with others | 3.87 | 0.169 |
| **Use of Mobility Aid** |  |  |
| Does not use mobility aid | *Ref* |  |
| Uses mobility aid | -6.31 | 0.058 |
| **Number of Non-ocular Comorbidities** | -0.00 | 0.994 |
| **Number of Ocular Comorbidities** | -6.24 | **0.042** |
| **Glaucoma** |  |  |
| No | *Ref* |  |
| Yes | 2.88 | 0.284 |
| **Retinal disease** |  |  |
| No | *Ref* |  |
| Yes | -7.57 | **0.004** |
| **Cataract** |  |  |
| No | *Ref* |  |
| Yes | 1.22 | 0.709 |
| **Socioeconomic status during COVID-19** |  |  |
| less than $10,000 | *Ref* |  |
| $10,001 - $25,000 | -6.54 | 0.356 |
| $25,001 - $50,000 | -4.64 | 0.508 |
| $50,001 - $75,000 | -0.29 | 0.967 |
| $75,001 - $100,000 | 5.53 | 0.484 |
| $100,001 - $125,000 | 3.57 | 0.659 |
| $125,001 - $150,000 |  |  |
| Greater than $150,000 | -0.50 | 0.954 |

Unadjusted effects of variables with depressive symptoms.

| **Variables** | **Coefficient** | **P-value** |
| --- | --- | --- |
| **Age** | -0.01 | 0.918 |
| **Education** |  |  |
| Completed high school or less | *Ref* |  |
| Completed more than high school | 1.98 | 0.149 |
| **Living Arrangement** |  |  |
| Home alone/ Nursing/Retirement home | *Ref* |  |
| Home with others | -0.05 | 0.974 |
| **Use of Mobility Aid** |  |  |
| Does not use mobility aid | *Ref* |  |
| Uses mobility aid | 4.35 | **0.016** |
| **Number of Non-ocular Comorbidities** | -0.00 | 0.994 |
| **Number of Ocular Comorbidities** | 2.10 | 0.227 |
| **Glaucoma** |  |  |
| No | *Ref* |  |
| Yes | -1.99 | 0.186 |
| **Retinal disease** |  |  |
| No | *Ref* |  |
| Yes | 2.74 | 0.066 |
| **Cataract** |  |  |
| No | *Ref* |  |
| Yes | -0.43 | 0.812 |
| **Socioeconomic status during COVID-19** |  |  |
| less than $10,000 | *Ref* |  |
| $10,001 - $25,000 | -4.42 | 0.286 |
| $25,001 - $50,000 | -3.13 | 0.445 |
| $50,001 - $75,000 | -4.51 | 0.278 |
| $75,001 - $100,000 | -1.24 | 0.788 |
| $100,001 - $125,000 | -8.00 | 0.093 |
| $125,001 - $150,000 |  |  |
| Greater than $150,000 | -3.42 | 0.503 |

Unadjusted effects of variables with anxiety symptoms.

| **Variables** | **Coefficient** | **P-value** |
| --- | --- | --- |
| **Age** | -0.01 | 0.688 |
| **Education** |  |  |
| Completed high school or less | *Ref* |  |
| Completed more than high school | -0.01 | 0.989 |
| **Living Arrangement** |  |  |
| Home alone/ Nursing/Retirement home | *Ref* |  |
| Home with others | 0.71 | 0.253 |
| **Use of Mobility Aid** |  |  |
| Does not use mobility aid | *Ref* |  |
| Uses mobility aid | 0.58 | 0.428 |
| **Number of Non-ocular Comorbidities** | 0.11 | 0.492 |
| **Number of Ocular Comorbidities** | 0.53 | 0.438 |
| **Glaucoma** |  |  |
| No | *Ref* |  |
| Yes | -0.12 | 0.838 |
| **Retinal disease** |  |  |
| No | *Ref* |  |
| Yes | 0.84 | 0.157 |
| **Cataract** |  |  |
| No | *Ref* |  |
| Yes | -0.71 | 0.328 |
| **Socioeconomic status during COVID-19** |  |  |
| less than $10,000 | *Ref* |  |
| $10,001 - $25,000 | -0.05 | 0.976 |
| $25,001 - $50,000 | 0.21 | 0.898 |
| $50,001 - $75,000 | 0.37 | 0.822 |
| $75,001 - $100,000 | -1.43 | 0.434 |
| $100,001 - $125,000 | -0.83 | 0.656 |
| $125,001 - $150,000 |  |  |
| Greater than $150,000 | -1.50 | 0.458 |

Unadjusted effects of variables with sleep quality.

| **Variables** | **Coefficient** | **P-value** |
| --- | --- | --- |
| **Age** | -0.01 | 0.831 |
| **Education** |  |  |
| Completed high school or less | *Ref* |  |
| Completed more than high school | 0.82 | 0.212 |
| **Living Arrangement** |  |  |
| Home alone/ Nursing/Retirement home | *Ref* |  |
| Home with others | -1.17 | 0.115 |
| **Use of Mobility Aid** |  |  |
| Does not use mobility aid | *Ref* |  |
| Uses mobility aid | 1.73 | **0.044** |
| **Number of Non-ocular Comorbidities** | 0.22 | 0.239 |
| **Number of Ocular Comorbidities** | 0.36 | 0.647 |
| **Glaucoma** |  |  |
| No | *Ref* |  |
| Yes | -0.56 | 0.417 |
| **Retinal disease** |  |  |
| No | *Ref* |  |
| Yes | 0.57 | 0.416 |
| **Cataract** |  |  |
| No | *Ref* |  |
| Yes | -0.28 | 0.747 |
| **Socioeconomic status during COVID-19** |  |  |
| less than $10,000 | *Ref* |  |
| $10,001 - $25,000 | -3.26 | 0.086 |
| $25,001 - $50,000 | -3.26 | 0.083 |
| $50,001 - $75,000 | -3.47 | 0.068 |
| $75,001 - $100,000 | -4.43 | **0.037** |
| $100,001 - $125,000 | -4.67 | **0.032** |
| $125,001 - $150,000 |  |  |
| Greater than $150,000 | -2.50 | 0.282 |

Unadjusted effects of variables with social support and community integration.

| **Variables** | **Coefficient** | **P-value** |
| --- | --- | --- |
| **Age** | -0.10 | 0.092 |
| **Education** |  |  |
| Completed high school or less | *Ref* |  |
| Completed more than high school | -1.23 | 0.169 |
| **Living Arrangement** |  |  |
| Home alone/ Nursing/Retirement home | *Ref* |  |
| Home with others | -0.67 | 0.509 |
| **Use of Mobility Aid** |  |  |
| Does not use mobility aid | *Ref* |  |
| Uses mobility aid | -1.22 | 0.297 |
| **Number of Non-ocular Comorbidities** | -0.50 | **0.047** |
| **Number of Ocular Comorbidities** | -1.51 | 0.173 |
| **Glaucoma** |  |  |
| No | *Ref* |  |
| Yes | 0.80 | 0.415 |
| **Retinal disease** |  |  |
| No | *Ref* |  |
| Yes | -3.06 | **0.001** |
| **Cataract** |  |  |
| No | *Ref* |  |
| Yes | -0.07 | 0.951 |
| **Socioeconomic status during COVID-19** |  |  |
| less than $10,000 | *Ref* |  |
| $10,001 - $25,000 | -0.02 | 0.993 |
| $25,001 - $50,000 | 0.33 | 0.900 |
| $50,001 - $75,000 | 0.13 | 0.962 |
| $75,001 - $100,000 | 3.38 | 0.254 |
| $100,001 - $125,000 | 0.92 | 0.762 |
| $125,001 - $150,000 |  |  |
| Greater than $150,000 | 1.67 | 0.610 |

**Appendix C.** Results of the Validation of Backwards Stepwise Linear Regression Models

1. 25-item National Eye Institute Visual Function Questionnaire model

Linearity:

Constant variance of residuals:

Normality of residuals:

Multicollinearity:


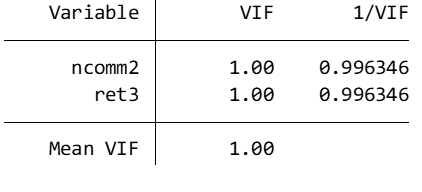


1. Center for Epidemiological Studies-Depression

Constant variance of residuals:

Normality of residuals:

Multicollinearity:

1. Hospital Anxiety and Depression Scale – Anxiety subscale

Constant variance of residuals:

Normality of residuals:

1. Pittsburgh Sleep Quality Index

Constant variance of residuals:

Normality of residuals:

1. Community Integration Questionnaire

Linearity:

Constant variance of residuals:

Normality of residuals:

Multicollinearity:

| **Model outcome** | **Root Mean Square Error** | **Mean Absolute Error** |
| --- | --- | --- |
| **NEI VFQ-25** | 10.98 | 8.98 |
| **CES-D** | 6.43 | 5.30 |
| **PSQI** | 3.02 | 2.56 |
| **HADS-A** | 2.62 | 2.18 |
| **CIQ** | 3.71 | 2.86 |

**Appendix D.** Backwards linear regression model assessments from leave-one-out cross validation.
